# Social dignity for marginalized people in public healthcare: an interpretive review and building blocks for a non-ideal theory

**DOI:** 10.1007/s11019-020-09987-8

**Published:** 2020-10-27

**Authors:** Jante Schmidt, Margo Trappenburg, Evelien Tonkens

**Affiliations:** Department Citizenship and Humanisation of the Public Sector, University of Humanistic Studies, P.O. Box 797, 3500 AT Utrecht, The Netherlands

**Keywords:** Social dignity, Public healthcare, Marginalized populations, Interpretive literature review, Non-ideal theory

## Abstract

Jacobson (Social Science & Medicine 64:292–302, 2007) finds two distinct meanings of “dignity” in the literature on dignity and health: (1) intrinsic *human* dignity and (2) *social* dignity constituted through interactions with caregivers. Especially the latter has been central in empirical health research and warrants further exploration. This article focuses on the social dignity of people marginalized by mental illness, substance abuse and comparable conditions in extramural settings. 35 studies published between 2007 and 2017 have addressed this issue, most of them identifying norms for social dignity: civilized interactions, non-stigmatizing treatment, treatment as unique individuals, being taken seriously, maintaining a positive identity, experiencing independence, relating to others, and participating in daily life. We argue that these norms belong to ideal theory, whereas we agree with Robeyns (Social Theory and Practice 34:341–362, 2008) and others that improving practice is better served by non-ideal theory. Towards this end, we derive from the literature four building blocks for a non-ideal theory of dignity: (1) avoid violations of dignity rather than seeking to promote it; (2) dignity is not a goal to be reached; it requires ongoing effort; (3) promoting dignity is a balancing act; contradictory norms can make it impossible to realize; and (4) dignity can be undermined by organizational and discursive constraints.

## Introduction

Concerns about dignity have been central in healthcare policies and research over the past decade, specifically in the realm of public health (Winter and Winter 2018). Mann already argued in 1997 that public health would benefit from analyses of the “burdens on dignity which constitute the societal roots of health problems” (2006/1997, p. 1940). Health and dignity often intertwine because the experience of dignity is contingent on both how people view themselves and on how others see them (Leget 2013; Mann 2006). When people are socially marginalized due to for example illness, substance abuse, poverty or homelessness, they are especially vulnerable to violations of their dignity. Ill health itself can undermine dignity by reducing control over one’s body, emotions and mental faculties, while the requirements of treatment may restrict one’s freedom (Jones 2015). Marginalization is also often accompanied by social stigma which negatively affects both mental and physical health (Link and Phelan 2006).

Despite the salience of dignity for people marginalized by their health or social status,^1^ dignity in public healthcare has received much less scholarly attention than in other areas such as end of life care (Chochinov 2002; Chochinov et al. 2005) or intramural care (Gallagher et al. 2008; Kane and De Vries 2017; Šaňáková and Čáp 2018). This article contributes to a non-ideal theory of dignity in public healthcare through an interpretative review of the empirical literature on the dignity of marginalized people as they receive treatment or support. We draw on meta-ethnography aimed at theory building (Dixon-Woods et al. 2006; cf. Brookfield et al. 2019; Flemming and McInnes 2012).

Our study builds on review articles by Jacobson (2007) and Leget (2013), both of whom point out that imprecise usage of the concept dignity has lead to the critique that the concept is vague and overly abstract. Jacobson notes that two distinct meanings of dignity prevail in the literature on dignity and health. The first is *human* dignity: “the inherent and inalienable value that belongs to every human being simply by virtue of being human” (2007, p. 294). The second is *social* dignity, which rests on “relational practices” (Leget 2013). For Jacobson, social dignity enacts “the abstract notion of human dignity in behavior, perception, and expectation”, “a contingent quality lost or gained within social interactions” (2007, p. 294). This distinction between human and social dignity is echoed in, for example, the distinction between absolute and relative dignity (Edlund et al. 2013), universal Kantian and aspirational dignity (Killmister 2010), and between universal human dignity (“*Menschenwürde*”) and three types of social dignity: dignity of merit, moral stature and identity (Nordenfelt 2004). Jacobson (2007) finds that empirical health research mostly pertains to social dignity—how dignity is maintained or threatened through social interactions in the context of care—and argues that enough is known about social dignity in general, so that “this work should now strive for greater explanatory power” by answering more specific questions, for example if “perceptions of social dignity are different for different populations” (p. 299).

Following Jacobson’s call for greater precision, our first theoretical step is to interpret “social dignity” for the specific context of public healthcare for marginalized people. Building on Leget’s claim that social dignity rests on “relational practices” (Leget 2013), we inductively examine which relational practices are deemed in the empirical literature to promote social dignity. We distil eight such relational practices from our dataset, for example that clients should be treated in non-stigmatizing ways and experience civilized interactions.

Few people would object to these practices and to the norms they entail; hardly anyone would argue that people *should* be treated in a stigmatizing way. Nevertheless, the violation of the dignity of marginalized persons is a serious problem in public healthcare, as the authors in our dataset convincingly show. Why does this remain the case if it is clear what the promotion of dignity looks like? We argue that the eight practices to promote dignity distilled from the dataset belong to the realm of ideal theory, by which we mean “utopian or idealistic theory” concerning a conception of a fully just society (Valentini 2012). Philosophers have argued that we need non-ideal theory alongside ideal theory for three reasons. First, non-ideal theory might more effectively support transitions from current realities to a fully just end-state. Second, non-ideal theory might help us better cope with partial compliance, while ideal theory presupposes full compliance. Third, non-ideal theory might provide better guidance in real life, as it considers constraints on feasibility as well as unintended consequences (Robeyns 2008; Valentini 2012). Our article seeks to contribute to a non-ideal theory of social dignity for this third reason.

Robeyns (2008) argues that philosophers need social scientists to investigate constraints on feasibility as well as the unintended consequences of any plan of action. Towards this end, social scientists can help philosophers distinguish between policy-induced circumstances that: (1) can be easily altered; (2) can only be altered at serious cost; and (3) are inherent to the human condition. Our second step in theory building thus entails examining the studies in our dataset to find out why, although few people would object to the eight practices of promoting dignity in public healthcare, violations of dignity remain serious problems. This enables us to construct four additional building blocks for a non-ideal theory of dignity in public health for marginalized people.

A non-ideal theory of social dignity is long overdue. More than a decade after the publication of Jacobson’s review—which argued that more specific questions about social dignity should be posed and answered—authors in the field continue to focus on general, ideal situations of promoting dignity by asking study participants how they define dignified treatment. With this article, we hope to open avenues for a non-ideal theory of dignity for marginalized people that will do justice to constraints on feasibility as well as to unintended consequences.

## Methods

### Search strategy and selection criteria

Our argument is based on an analysis of the empirical literature on social dignity in public healthcare for marginalized people. Formulating questions and selecting and extracting data was an iterative process (Dixon-Woods et al. 2006). To arrive at our dataset, we used the search term “dignity” pertaining to all fields and then applied the following selection criteria: (1) the article studies care and support for people marginalized due to their health and/or social status (e.g. because of illness, substance abuse, poverty or homelessness) in an extramural or semi-mural setting, focusing on the perspectives of care recipients and/or caregivers. Studies with another central concept such as “stigma” or “recognition” were included so long as it was explicitly linked to dignity; (2) the study is based on empirical data, clearly recognizable in the text and not consisting of a single case; (3) the research took place in an OECD country; and (4) the article is written in English.

**Fig. 1:**
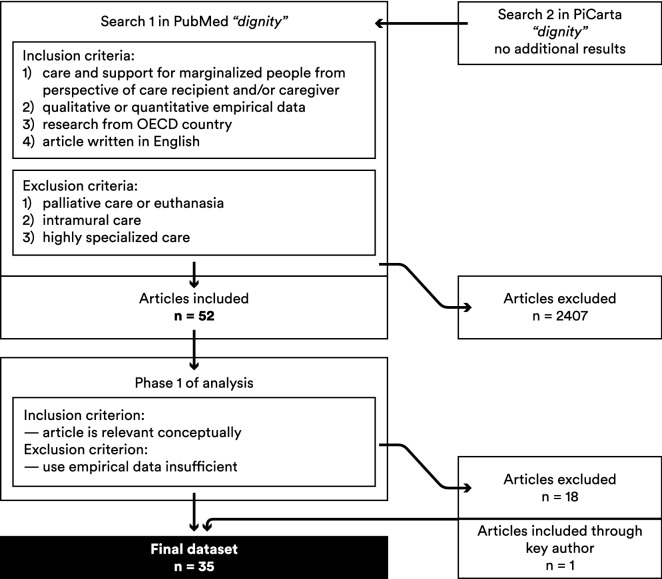
Flow chart searching process

We excluded articles on palliative care, euthanasia, intramural care and highly specialized care, for example for cancer and dementia. As these fields are prominent in the research on dignity and health, many studies were excluded (n = 2407, see Fig. 1). The exclusion criteria were refined in the process of selection. Quantitative studies in which dignity was among the variables measuring something else, for example “responsiveness”, were not included as they did not add to our theoretical insight. As a result, no purely quantitative studies were included in the final dataset.

### Search outcomes and analysis

As this article builds on Jacobson’s review (2007), we demarcated the search period as 2007 to March 2017. We ran our search in PubMed (Medline/PMC), which resulted in 2459 hits. A second search in the Dutch national catalogue of university libraries PiCarta generated no new results. Additional articles were identified through publications of key author Jacobson (n = 1). The first selection was made on the basis of titles and/or abstracts by the first two authors (JS, MT) working closely together and refining the selection criteria; 52 articles were selected. The second selection was made after the first phase of analysis and resulted in the final dataset of 35 studies (see Fig. 1).

Each article was summarized by looking at the research question/aim, methods and (number of) study participants. We recorded the definition of dignity, if any, and described the rationale for research. We included the main findings regarding dignity, consisting of the authors’ findings and participants’ verbatim quotes. We stayed close to the data by sticking with the words used by the authors. At the same time, we started to voice our own thoughts and questions separately, discerning overarching themes regarding social dignity. As these themes developed, we designed a second tool for analysis consisting of the following questions: Is dignity also or mainly described in terms of violating dignity? What, according to the authors, is/can be done to advance or restore dignity? What do the authors say about the feasibility of advancing or restoring dignity?

All articles were analysed by the first author and by either one or both of the other authors. Our dataset is presented in Table 1. All articles are numbered and will hereafter be referred to by their numbers.

**Table 1:** Characteristics of studies included in synthesis

Author	Year	Country	Participant group and number of participants	Type of data and data collection method	Research question or research aim
1. Baillie and Gallagher	2011	United Kingdom	51 nurses actively involved in the Royal College of Nursing”dignity campaign”	Qualitative data; semi-structured interviews	Discuss one theme from the study’s results that illuminate nurses’ strategies to respect dignity in care across diverse care settings
2. Bossy et al	2017	Norway	16 diabetes type 2 patients; part does and part does not participate in self-help group	Qualitative data; mixed focus groups	How do individuals with type 2 diabetes understand how group-based self-management support may (or may not) help accommodate the challenges of living with a long-term condition? why do some join while others refuse to participate in group-based self-management support?
3. Bramesfeld et al	2007	Germany	50 mental health service users	Qualitative data; focus groups	Does the WHO responsiveness concept with its eight domains [one of which is dignity] appropriately reflect the non-medical expectations of mental healthcare users?
4. Cairns et al	2013	United Kingdom	192 health and social care professionals providing care for older people	Qualitative data and quantitative data; survey, both closed and open-ended questions	What does dignified care mean for health and social care professionals? what are the most important aspects of dignified care as perceived by health and social care professionals?
5. Caspari et al	2013	Norway	17 caretakers (e.g. nurses, physiotherapists, psychologists) working in rehabilitation wards for patients suffering from head injuries or Multiple Sclerosis	Qualitative data; focus groups	Explore health personnel’s views on dignity in rehabilitation institutions and how they attend to, preserve and promote the dignity of patients who suffer from head injuries or Multiple Sclerosis
6. Collins et al	2008	United States	24 Latina women (“identified themselves as Hispanic, Spanish or Latina”) with severe mental illness	Qualitative data; qualitative interviews	How do the intersections of race/ethnicity, gender, class, status and the stigma of mental illness influence women’s sexual relationships and HIV risk?
7. Cook and Nunkoosing	2008	Australia	12 impoverished older persons; clients of the same social service agency	Qualitative data; narrative interviews	Examine the legitimacy and inadvertent consequences of paid-for participation (participants were paid 20 dollars for an interview)
8. Greer et al	2016	United States	29 adult food pantry patrons	Qualitative data; focus groups	What individual, interpersonal, organizational, environmental, and policy factors influence food pantry patrons’ ability to acquire sufficient food in a large, urban area?
9. Hedman Ahlström et al	2010	Sweden	8 parents identified with major depression	Qualitative data; narrative interviews	Elucidate the meaning of major depression in family life from the viewpoint of the ill parent
10. Holm and Severinsson	2014	Norway	6 care professionals: 4 mental health nurses, 1 geriatric nurse and 1 physiotherapist	Qualitative data; focus groups	Explore healthcare team members’ reflections on the ethical dilemmas involved in promoting self-management among depressed older persons
11. Holm et al	2014	Norway	15 elderly persons diagnosed with a depressive or mood disorder	Qualitative data; in-depth interviews	Deepen the understanding of depressed elderly persons’ lived experiences of physical health problems
12. Hughes et al	2008	United States	10 impoverished adults with advanced AIDS	Qualitative data; group interviews	Describe the meaning and experience of dignity to the urban poor with advanced AIDS, receiving care in an AIDS-dedicated nursing home unit
13. Jacobson	2009 a	Canada	64 participants including marginalized people, individuals who provide health or social services to these populations, and people working in the field of health and human rights	Qualitative data; semi-structured interviews	Develop a taxonomy of dignity – “a coherent vocabulary and framework to characterize dignity”
14. Jacobson	2009 b	Canada	64 participants including marginalized people, individuals who provide health or social services to these populations, and people working in the field of health and human rights	Qualitative data; semi-structured lived experience interviews	Understand the violation of dignity in healthcare and explore the context in which such violations take place
15. Jacobson et al	2009	Canada	64 participants including marginalized people, individuals who provide health or social services to these populations, and people working in the field of health and human rights	Qualitative data; semi-structured interviews	Examine the city as a setting for dignity encounters, seeking to understand how urban geography figures in the social processes that violate or promote dignity
16. Jacobson and Silva	2010	Canada	64 participants including marginalized people, individuals who provide health or social services to these populations, and people working in the field of health and human rights	Qualitative data; semi-structured lived experience interviews	Look at the ways in which the notion of dignity promotion can be used to complement our understanding of the principle of beneficence
17. Johnston et al	2015	Canada	21 adults with physical disabilities who visit a fitness centre adapted to people with disabilities	Qualitative data; focus groups and one-on-one interviews, visual images (participants made photos to illustrate experiences of dignity) and field notes	Understand the meaning of dignity and its importance to exercise participation
18. Kinnear et al	2014	United Kingdom	81 health and social care professionals who provide care for older people	Qualitative data; focus groups and qualitative interviews	What does dignified care mean for health and social care professionals?
19. Kinnear et al	2015	United Kingdom	192 health and social care professionals who provide care for older people	Qualitative data and quantitative data; self-completion questionnaire, both closed and open-ended questions	How dignified care for older people is understood and delivered by health and social care professionals; how organisational structures and policies can promote and facilitate, or hinder, the delivery of dignified care
20. Klingeman	2017	Poland	112 participants, staff (43) and patients (69) of drug treatment facilities (three outpatient, three inpatient, three substitution and three harm reduction programs)	Qualitative data; focus groups interviews	Describe experience of equality in the dignity and rights of patients in four different specialist drug treatment settings
21. Kohon and Carder	2014	United States	47 low-income adults (55 +) currently living in or on the waiting list for publicly subsidized housing	Qualitative data; auto-photography with narrative interviews and photo elicitation with photographs taken by research participant	How do low-income older adults living in, or on a waiting list for, subsidized housing perceive their health and independence?; how do low-income older adults describe the benefits and challenges associated with their current housing or surrounding neighbourhood?; how does living in or applying for subsidized housing affect older adults’ identity and sense of self?
22. Lee	2012	United-States	12 mothers residing in an urban homeless shelter with their children	Qualitative data; observation and interviews (“ethnonursing research method”: focuses on naturalistic, open, and inductive modes of knowledge discovery)	Identify the meanings and experiences of family homelessness and its relationship to health as expressed by Appalachian mothers
23. Lindgren et al	2015	Sweden	11 adults (18 +) who had participated in medication-assisted treatment for opioid dependency for more than 3 years	Qualitative data; narrative interviews	Describe the lived experiences of participating in a medication-assisted treatment as disclosed by individuals with opioid dependence
24. Melin et al	2017	Sweden	13 adults (18 +) who had participated in medication-assisted treatment for opioid dependency for more than 3 years	Qualitative data; narrative interviews	Describe experiences of living with opioid dependence
25. Moe et al	2013	Norway	11 elderly patients (80 +) of home nursing care	Qualitative data; narrative interviews	Illuminate the meaning of receiving help from home nursing care for chronically ill, elderly persons living in their homes
26. Narayan et al	2013	Australia	5 adolescents (all female) in hospital setting; 12–16 years of age	Qualitative data; interviews 15–30 min	How do young people in a children’s hospital perceive dignity and how do their perceptions of dignity impact on their healthcare experience?
27. Ørjasæter and Ness	2017	Norway	12 psychiatric patients	Qualitative data; in-depth interviews	What enables meaningful participation in a music and theatre workshop, located in a mental health hospital, from a first-persons perspective of people with long-term mental health problems?
28. Radley et al	2016	Scotland	41 participants who either received opiate replacement therapy (ORT) or were the caregiver of someone prescribed ORT	Qualitative data; focus groups	Explore experiences of service users attending a community pharmacy to receive opiate replacement therapy
29. São José	2016	Portugal	49 participants: 24 elderly (65 +) receiving home care, 8 (female) home care workers and 17 family caregivers	Qualitative data; participant observation and informal conversations	Explore how older individuals receiving social care in the community, specifically home care, experience the loss of dignity and how they preserve their dignity
30. Skar and Soderberg	2012	Sweden	9 men who have personally filed a complaint with the Patients’ Advisory Committee in the county council	Qualitative data; semi-structured interviews	Describe experiences of dissatisfaction with encounters in healthcare among men who personally filed a complaint to the Patients Advisory Committee in the county council
31. Skorpen et al	2016	Norway	13 relatives of patients with psychoses, recruited via relatives’ user organisations	Qualitative data; Q-methodology, 51 statements (concourse based on 17 qualitative interviews with patients, staff members and relatives)	Reveal relatives’ opinions regarding what is important for taking care of patients’ dignity
32. Werner and Malterud	2017	Norway/Denmark	9 adults who grew up in families with problem-drinking parents	Qualitative data; qualitative interviews (retrospective accounts)	Explore informal adult support experienced by children of parents with alcohol problems during childhood and adolescence, to understand how professionals could show them recognition in a similar way
33. Whitaker et al	2011	Ireland	35 drug users who were engaging in or who had engaged in sex work (31 women and 4 men)	Qualitative data; in-depth interviews	Present selected findings on stigma experienced by drug-using sex workers in Dublin
34. Whitley and Campbell	2014	United States	People with severe mental illness (SMI) living in small scale congregate housing units meant for people recovering from SMI; “recovery communities” [exact number of participants is not given]	Qualitative data; 22 focus groups over a period of 5 years, augmented by participant observation (authors state: “most residents participated repeatedly in multiple groups over time […] Focus groups typically included about six to twelve residents, although exact numbers were difficult to ascertain as many participants came and went during the group”)	Document and analyse strategies of management and control of stigma in a sample of people recovering from severe mental illness
35. Wiklund	2008	Sweden	9 individuals with rich experience of drug addiction	Qualitative data; narrative interviews	Describe caring needs associated with existential aspects of living with addiction

## Results

### Reviewed studies

Table 1 shows the characteristics and research questions/aims of the 35 studies in our dataset. The clients’ perspective is central in 21 studies; six studies take the perspective of care professionals and one study focuses on clients’ relatives. Seven studies combine these perspectives. Studies were performed in eleven different countries, the highest number (n = 8) in Norway.

Participant groups include marginalized older people (7; 11; 21; 25; 29), people with mental health problems (3; 27; 6; 9; 34), clients with a history of drug abuse (33; 35) and clients involved in drug treatment programs (20; 23; 24; 28). Caregivers include health and social professionals caring for older people (4; 10; 19) and professionals working at, for example, a rehabilitation ward (5) or a drug treatment facility (20). The relatives include family members of patients with psychoses (31) and family caregivers of older people (29).

Studies in our dataset made use of qualitative interviewing (n = 19), focus groups (n = 10), observation (n = 1), a combination of these (n = 2), Q-methodology (n = 1), and open ended questionnaires (n = 2).

### Relational practices that promote social dignity

Most articles use dignity as a tool to criticize prevailing care practices. Authors either suspect a lack of dignity in the care setting and study its meaning for affected people, or find that (lack of) dignity is central to the experiences described by study participants, making dignity a category of analysis. Most authors do not define dignity beforehand.

We categorized the relational practices in which social dignity is or could be promoted under eight main headings. Findings related to the organization of physical space in the care environment are not included. We distinguish between four types of client–caregiver interaction: (1) civilized interactions; (2) being treated in a non-stigmatizing way; (3) being treated as a unique individual; and (4) being taken seriously/listened to. We further discern four aspects that bear on the client’s social position: (1) maintaining a sense of positive identity; (2) experiencing independence/autonomy; (3) relating to others; and (4) participating in daily life. The results are summarized in Table 2.

**Table 2:** Relational practices that promote social dignity, sorted by main themes of each article

Relationship between client and caregiver	
Civilized interactions	3; 8; 12; 13; 14; 16; 18; 25; 30; 33
Being treated in a non-stigmatizing way	3; 8; 11; 12; 13; 14; 15; 16; 20; 22; 24; 27; 28; 33
Being treated as a unique individual	1; 3; 4; 5; 13; 14; 16; 18; 19; 25; 27; 30; 31
Being taken seriously/listened to	3; 5; 10; 11; 13; 14; 16; 27; 30; 31; 32; 35
Social position of the client	
Maintaining a sense of positive identity	2; 6; 7; 9; 13; 15; 17; 21; 22; 26; 29; 34; 35
Experiencing independence/autonomy	2; 5; 13; 14; 15; 17; 26
Relating to others (peers, family members)	2; 9; 12; 17; 23; 27; 29; 34; 35
Participating in daily life	9; 13; 17; 23; 27; 29; 34

In Table 2, the studies are sorted according to the main theme(s) surrounding dignity advanced by their authors. First, civilized interactions between client and caregiver concern attentive and timely communication. For example: “It starts with the people at reception. They should try to look you in the eye if possible and really take notice of you and not just be occupied with their desk when dealing with you” (3: 885). People should be addressed in the way they prefer, formally when desired (12; 14; 18).

Second, the stigmatization of clients can stand in the way of civilized interactions. Clients can, for example, feel patronized by caregivers (8; 12; 13; 14; 16; 31). In study 8, participants felt that they were “talked down to”, “like I’m the lowest of the low” in interactions with staff (p. 201). Caregivers thus need to avoid stigmatizing clients. Caregivers can also counter the stigma people have endured in society or at the hands of other caregivers by treating them in non-stigmatizing ways. Study 24 shows how care professionals supported the recovery of people who have been treated for opioid dependence by treating them in a “non-judgmental and respectful manner”: even when they relapse, they stay with their clients and do not lose faith in them.

Third, people want to be treated as unique individuals with specific values and needs (16; 31). It is important that caregivers look beyond the client’s diagnosis. Study 27 finds that psychiatric patients, when participating in music and theatre workshops, “feel seen, met, and understood as whole human beings” (p. 1604) rather than as mere patients who are ill.

Fourth, when a person is exclusively approached in terms of a diagnosis, this results in a feeling of not being taking seriously (3; 5). Study 11 describes the issue of “diagnostic overshadowing” where the physical complaints of depressed older people were deemed imaginary and rooted in depression rather than taken seriously. Other studies also show that clients feel their dignity to be undermined when healthcare providers do not take their symptoms seriously (13; 14; 30).

Marginalized people must cope with the restrictions their health places on them as well as the social stigma that comes with their condition. One way to achieve dignity is by maintaining a positive sense of identity, for example “the construction of an identity as worthy individuals despite the stigma associated with type 2 diabetes” (2: 164). Other practices bearing on the client’s social position—experiencing independence/autonomy, participating in daily life and relating to others—also contribute to a positive sense of identity. Study 15 discusses the importance of participants having a sense of autonomy; a formerly homeless participant states: “That is where dignity begins, a place of your own, a home” (p. 729). A home offers privacy and control but also a sense of normality: “I don’t know, you just feel like everyone else then, you know?” (p. 729). In study 23, people with opioid dependence undergoing medication-assisted treatment claim they now live a “life in dignity” with a functioning social network, a job and a house. Participation in daily life also concerns relating to others, and participants felt they now had a chance to make amends with relatives and friends. One participant stated: “I feel like a normal human being, I can meet ordinary people and talk to them” (p. 968). Relating to others in the same marginalized social position could also help to maintain a positive sense of identity in the face of stigmatization (2; 12; 17; 34).

The eight relational practices that promote social dignity found in the literature can be understood as an operationalization of social dignity. This constitutes our first step in theory building. To study the promotion of social dignity among marginalized people in public healthcare, scholars no longer have to start from scratch, although many of the studies in our dataset still do so. They can build on these eight practices found to constitute social dignity in this context.

Nevertheless, these eight practices all concern ideal situations and are therefore part of ideal theory. In the remainder of this article, we move from ideal to non-ideal theory. We now reconsider the studies in our dataset, asking the following questions: If caregivers and care recipients seem to agree that these eight practices are important, why do they not always act accordingly? Why is social dignity so difficult to attain, when it is so clear what should be done to promote it? Our studies provide only partial answers to these questions. What follows should thus also be read as our interpretation of this untold part of the story.

### Four building blocks for non-ideal theory

#### Promoting social dignity starts by identifying violations of dignity

First, as Margalit (1996) argues, dignity is harder to pinpoint than its opposite: the violation of dignity. Our data underline this point: social dignity is usually understood in terms of countering its violation. This violation is often termed stigmatization (e.g. in studies 6, 23, 33, 34), but sometimes “suffering” (9) or “intrusion” (10). Study 17, on adults with physical disabilities who visit an adapted fitness centre, found that they “present dignity as a taken-for-granted construct that only comes to awareness when it is threatened” (p. 107). Participants defined dignity negatively as “an area where you’re not criticized for what you’re trying to do… You’re allowed to voice your opinion without ridicule” (p. 114).

There is thus an asymmetry in the vocabulary concerning dignity on the one hand and the violation of dignity on the other. Participants have more words to describe instances of dignity being violated. Authors regularly emphasize this imbalance. In study 3, “Two-thirds of expectations in the category dignity were expressed through negative examples showing how people do not want to be treated” (p. 885). Study 5 notes “how easily concepts relating to the opposite of ‘dignity’ were mentioned” (p. 2321). Study 19 reflects on “the greater emphasis in our participants’ narratives on the barriers as opposed to the supportive factors [of dignity]” (p. 831). Dignity seems to be best studied through experiences of its violation. This, then, is our first principle for building a non-ideal theory on dignity for marginalized people: start by identifying the negative. People may not always be able to explain what would further their dignity, but they do know how it feels to be ridiculed, ignored, or stigmatized.

#### Promoting social dignity requires dignity work

When participants are prompted to think about times when they experienced enhanced dignity, they talk about the promotion of dignity as a distinct, effortful activity. Study 16 describes “dignity work” as “a deliberate attitude, behavior, or action engaged by an identifiable actor with the aim of creating, maintaining, defending, or reclaiming dignity” (p. 367). Dignity work can be performed by individual and collective agents to promote either their own dignity^2^ or the dignity of others^3^.

The concept of dignity work underlines that the promotion of social dignity takes time and effort, with no guarantee of success. For example, study 6 shows how female participants resist the stigma attached to mental illness in their communities by “aligning themselves with identities that bestow dignity and respect” (p. 395); the respectable identity of the “good girl”, although out of reach, plays a big role for them. Study 34 shows that participants with severe mental illness put great effort into avoiding stigma and gaining dignity by acting and appearing “normal”. These examples show that dignity work is often costly and its success sometimes limited. The costs of dignity work can also affect others. Study 29 finds that for older people receiving social care in the community, “preserving dignity is a process of struggle and resistance” (p. 339). In trying to maintain their activities, relationships and family roles, they can be demanding, complain and accentuate status, resulting in angry behavior towards family caregivers. Focusing on dignity work by care recipients and caregivers can unveil the practices that promote social dignity. Caregivers often invest great effort in approaching their clients in a civilized way, trying to avoid stigmatization and taking them seriously as unique individuals.

The second building block of our non-ideal theory of dignity for marginalized people is: maintaining and achieving dignity is a perpetual process that requires vigilance and effort.

#### Promoting social dignity is a balancing act

Promoting social dignity is riddled with tensions; a practice that protects or promotes social dignity may threaten it too. Study 9 shows that parents with major depression find dignity in “maintaining parenthood”—involving both joy in children and parenting as well as frustrations and disappointments in their own behaviour which challenges their sense of dignity. While the intensity of participation in daily family life must be adjusted, being a “good enough parent” remains a source of social dignity.

Treatments for clients that contribute to social dignity are also often imperfect as they sometimes both enhance and threaten dignity. Study 23 describes medication-assisted treatment for people with opioid dependence as a chance to live a life with dignity. But participants also suffer “double stigma” as society looks askance at both people with opioid dependency and medication-assisted treatment.

Ways of promoting social dignity can be at odds with one another, with the literature revealing a particular tension between maintaining autonomy and paternalism. Although autonomy is often seen as a crucial aspect of dignity, in some situations such as acute psychosis or severe dementia, others may need to make decisions for the client. Study 31 shows that some relatives of patients with psychoses emphasize how asymmetry in relationships between patients and staff negatively affects dignity; others point out that “taking care of a patient’s dignity sometimes involves taking total responsibility for a patient’s situation” (p. 126). Study 26 found that “challenging power structures and their own relative powerlessness was identified as important for protecting a vulnerable sense of personhood” for adolescents, who “viewed authoritarianism as a violation of dignity and need staff to join them in a balancing act between long-term medical benefit and promoting their developing self-concept” (p. 894). Guaranteeing clients the opportunity to make autonomous choices and caregivers sometimes over-ruling these choices to promote clients’ well-being can both promote dignity. Such tensions shed light on the dilemmas caregivers face in everyday care^4^.

The third building block of our non-ideal theory emphasizes that promoting dignity is a balancing act. This can particularly be the case in public healthcare when there is tension between the well-being and autonomy of individual clients and the well-being of the wider population (the literature we studied paid scant attention to this, with authors focusing on individuals). This tension must be recognized in any non-ideal theory of social dignity for marginalized people. Sometimes it is possible to strike the right balance between two evils; at other times, we need to acknowledge that furthering certain aspects of dignity comes at the price of sacrificing other aspects of dignity or the interests of other people. Non-ideal theory should acknowledge such perpetual dilemmas.

#### Promoting social dignity is restricted by organizational and discursive constraints

Finally, we need to consider the non-ideal circumstances in which social dignity takes shape, including the organizational constraints under which care and support institutions operate. Study 25 argues for holistic care to promote older people’s dignity but notes: “Nurses’ working days are busy, and there is no indication that this is going to change.[…] A culture of care should […] result in focusing on the person for the few minutes that the visit lasts” (pp. 745–746). Study 19 shows that most barriers to providing dignified care concern inter-linked issues of staffing, time and work pressure. The authors point out that “understanding the delivery of dignified care requires a broader focus than individual staff […]” (p. 839). Study 14 discerns two main institutional conditions that inform violations of dignity in healthcare: asymmetrical relationships between actors (e.g. differences in knowledge, priorities and power) and the harsh circumstances of healthcare settings (e.g. shortage of space, materials and time).

Second, we need to consider the macro societal context of dominant social norms and value systems. Study 14 points to “a social order of inequality”—forms of oppression such as sexism, racism and structural violence directed against the poor that can be reproduced in healthcare (p. 1543). Study 2 interprets the experiences of people with type 2 diabetes within the prevailing ideology of neoliberalism, in which responsibility for health is placed on the shoulders of individual citizens. Study 33 looks at the way drug-using sex workers experience stigma and suggests that “the government’s efforts to reduce harm might be unwittingly hampered by service providers, because the taken-for-granted language they use assigns drug users to a ‘dirty’ category” (p. 1097). Finally, study 6 shows how the stigma of severe mental illness places female participants in conflict with gender norms in their communities.

The fourth building block of our non-ideal theory is to consider how non-ideal organizational and discursive contexts constrain what is feasible. Social norms and organizational realities, including time and budget constraints, can restrict or promote social dignity. Caregivers and clients must cope with these restrictions.

## Discussion

This study aims to contribute to a non-ideal theory of social dignity. To encourage the much-needed transition from the abstract ideal of respect for human dignity to a non-ideal theory of social dignity that can guide action and (policy) implementation (Robeyns 2008), we have operationalized social dignity for the specific context of public healthcare for marginalized people. From the empirical literature we derived eight relational practices that promote social dignity in public healthcare for this target group. People must be (1) treated in a civilized way, (2) not be stigmatized, (3) seen as unique individuals who (4) are taken seriously and listened to. They must also be able to maintain (5) a positive sense of identity (6) by experiencing independence/autonomy, (7) by relating to others, and (8) by participating in daily life.

We then identified four building blocks for a non-ideal theory of social dignity: (1) furthering dignity should start by identifying violations of dignity; (2) promoting dignity requires intensive dignity work; (3) promoting dignity is a balancing act, and striking the right balance is not always possible; and (4) furthering dignity is limited by organizational and discursive constraints. Identifying these constraints should help to illuminate why, even when the relational practices that promote dignity are known, dignity in public healthcare is difficult to achieve.

The literature we analysed to construct our argument has some limitations. We compared studies from different countries, set in different kinds of institutions and among different groups of clients. The findings of these mostly inductive studies are reported using different words and analytical distinctions, which introduces some uncertainty into interpretation. Nevertheless, we think that the picture derived from our dataset is clear enough to serve as input for our non-ideal theory of social dignity.

Where do we go from here? Moral philosophers might closely consider the building blocks of the non-ideal normative theory we have developed thus far. For social scientists, the current theory should give shape to future questions for empirical research. We will briefly try to do both in this discussion by returning to the four building blocks.

As Robeyns (2008) suggests, the building blocks of non-ideal theory may help normative philosophers to distinguish between conditions and circumstances that can be altered, and conditions and circumstances that must be accepted and dealt with as best we can. The first building block mostly concerns strategy. Researchers, caregivers and policymakers who want to promote dignity for marginalized people should start by identifying and possibly avoiding violations of their dignity. Lowering their ambitions may help them formulate feasible goals in social policy. The second and third building blocks also temper ambitions: the second emphasizes that dignity work for marginalized people is an ongoing project for both clients and caregivers. The third shows that certain trade-offs are unavoidable.

The first three building blocks seem to be rooted in, if not the human condition, then the particular condition of marginalized people. This differs from the fourth building block concerning organizational and discursive constraints. Organizational, financial and time constraints are in principle amenable to change. For scholars and philosophers engaged in non-ideal theory, this encourages greater attention to the structural changes that would be needed to remedy organizational and discursive constraints. It would also be wise to not think too superficially about what change would require. Non-ideal theory must consider that society is intertwined in numerous ways; simply advocating for more resources to care for marginalized people would entail reduced budgets elsewhere—for environment? education? culture?—higher taxes, or both. While this may be warranted, non-ideal theorists should be aware of the costs involved.

For social scientists, we think it is no longer necessary or helpful to let their research be guided by general open questions such as what care recipients and caregivers understand dignity to be, and what it means for them to be treated in a dignified manner. The eight practices we identified already answer such questions, while our building blocks for non-ideal theory can help future scholars frame more specific questions. They may ask, for instance: How is the social dignity of clients violated in relationships of care, in relations with significant others (family, friends, peers), and in wider society (building block 1)? How do clients and caregivers work to protect or promote the social dignity of clients and caregivers (building block 2)? How do these same practices also run the risk of violating social dignity (building block 3)? How do organizational features and social norms restrict or promote social dignity (building block 4)?

Asking and answering such questions may well take social scientists back to moral philosophy to further specify a non-ideal theory of social dignity for marginalized people, which should be a collective endeavour between normative philosophers and social scientists. Hopefully such a realistic theory could truly help caregivers, policymakers and marginalized people who inhabit a non-ideal world.

## Footnotes

1 For concision, we hereafter refer to this group as “marginalized people”; we always mean people who are marginalized by society because of their health and/or social status.

2 Through contribution, creativity or resistance.

3 Through courtesy, recognition, acceptance, generosity, presence, love, advocacy, leveling and empowerment.

4 See Holm and Severinsson (2014) for how care professionals see the tension between paternalism and autonomy.
